# TREC Based Newborn Screening for Severe Combined Immunodeficiency Disease: A Systematic Review

**DOI:** 10.1007/s10875-015-0152-6

**Published:** 2015-04-17

**Authors:** Jet van der Spek, Rolf H. H. Groenwold, Mirjam van der Burg, Joris M. van Montfrans

**Affiliations:** 1 Department of Pediatric Immunology and Infectious Diseases, University Medical Center Utrecht, Lundlaan 6, PO Box 85090, 3508 AB Utrecht, The Netherlands; 2 Julius Center for Health Sciences and Primary Care, University Medical Center Utrecht, Utrecht, The Netherlands; 3 Department of Immunology, Erasmus MC, University Medical Center Rotterdam, Rotterdam, The Netherlands

**Keywords:** Severe Combined Immunodeficiency, T-cell lymphopenia, newborn screening, T-cell receptor excisions circles, screening algorithm, systematic review

## Abstract

**Background:**

Newborn screening (NBS) by quantifying T cell receptor excision circles (TRECs) in neonatal dried blood spots (DBS) enables early diagnosis of severe combined immunodeficiency disease (SCID). In recent years, different screening algorithms for TREC based SCID screening were reported.

**Purpose:**

To systematically review the diagnostic performance of published algorithms for TREC based NBS for SCID.

**Methods:**

PubMed, EMBASE and the Cochrane Library were systematically searched for case series and prospective cohort studies describing TREC based NBS for SCID. We extracted TREC content and cut-off values, number of retests, repeat DBS and referrals, and type and number of typical SCID and other T cell lymphopenia (TCL) cases. We calculated positive predictive value (PPV), test sensitivity and SCID incidence.

**Results:**

Thirteen studies were included, re-confirming 89 known SCID cases in case series and reporting 53 new SCID cases in 3.15 million newborns. In case series, the sensitivity for typical SCID was 100 %. In the prospective cohort studies, SCID incidence was ~1.7:100,000, re-test rate was 0.20–3.26 %, repeat DBS rate 0.0–0.41 % and referral rate 0.01–1.35 %. PPV within the five largest cohorts was 0.8–11.2 % for SCID and 18.3–81.0 % for TCL. Individual TREC contents in all SCID patients was <25 TRECs/μl (except in those evaluated with the New York State assay).

**Conclusions:**

The sensitivity of TREC based NBS for typical SCID was 100 %. The TREC cut-off score determines the percentage of non-SCID TCL cases detected in newborn screening for TCL. Adapting the screening algorithm for pre-term/ill infants reduces the amount of false positive test results.

**Electronic supplementary material:**

The online version of this article (doi:10.1007/s10875-015-0152-6) contains supplementary material, which is available to authorized users.

## Introduction

Severe combined immunodeficiency disease (SCID) is a group of inherited immunodeficiencies characterized by T cell lymphopenia (TCL). SCID is recognized as a pediatric emergency because it leads to severe and recurrent infections and is fatal within the first two years of life when left untreated [1–3]. Data collected by the Primary Immune Deficiency Treatment Consortium demonstrate survival rates of 94 % for those treated with hematopoietic stem cell transplantation in the first 3.5 months of life, 90 % in older infants without infections, and 82 % in those with resolved infections. In contrast, survival of infants older than 3.5 months with active infection during transplant was only 50 %, indicating that early diagnosis of SCID improves outcome [4].

SCID can be detected early by quantifying T-cell receptor excision circles (TRECs) in Guthrie card dried blood spots (DBS) using a real time quantitative polymerase chain reaction (RT-qPCR) [5]. Since TRECs are a DNA byproduct of T cell receptor recombination, low TRECs reflect TCL [6]. Hence, neonatal TREC levels can be used to detect impaired T cell development and thus to screen for SCID. After a positive screen, diagnostic follow-up is required to determine whether the patient suffers from typical SCID or another form of TCL and to establish a specific genetic diagnosis. The severity of SCID related symptoms, the improved outcome after early diagnosis, the availability of a screening test in combination with evidence that newborn screening (NBS) for SCID is cost-effective [7, 8] demonstrate that SCID fulfills the Wilson-Jungner screening criteria [9] and identify SCID as a suitable candidate for NBS.

In 2005, Chan and Puck proposed TREC measurement for NBS for SCID [5]. A NBS program for SCID using the TREC assay was successfully initiated in the state of Wisconsin [10]. This pilot study contributed to the unanimous recommendation of the US Secretary’s Advisory Committee on Heritable Disorders in Newborns and Children to add SCID to the Recommended Uniform Newborn Screening Panel in 2010. Since then, NBS for SCID has been implemented in an increasing number of states in the USA [7, 11]. Implementation of NBS for SCID outside the USA is upcoming: pilot studies have been conducted [12–18] and calls for implementation have been published [19–21] in Europe, Asia and South America.

Screening strategies adopted in different states and countries show heterogeneity. This complicates decision-making on the design of a uniform algorithm to use in countries that prepare to implement NBS for SCID. In general, screening algorithms start with the quantification of TREC content using RT-qPCR. Several screening programs measure a housekeeping gene (β-actin or RNaseP) in parallel in this initial test. An abnormal value results in re-testing of the initial DBS, while failure of control gene DNA amplification leads to a request for repeat DBS collection. All algorithms end with the possibility of referral for diagnostic evaluation [22]. However, TREC cut-off values, handling of equivocal or inconclusive results, and handling of subgroups (e.g., infants born prematurely) vary among different screening sites.

As more states and countries adopt TREC based NBS, an increasing number of screening results is published. Questions that arise from examining results of individual screening sites include how the TREC content of patients detected at different screening sites relates to the established diagnosis and local cut-off values, and how the USA data compare to data generated outside the USA.

To investigate the difference in diagnostic performance of the screening strategies used at various screening sites, we conducted a systematic review on TREC based NBS for SCID. We evaluated the diagnostic performance of the individual algorithms based on the number of retests and repeat samples required, the number of referrals and the positive predictive value (PPV) for SCID. The number of retests and repeat samples are significant measures of performance, since they reflect the burden of additional testing exerted on healthy individuals and the health care system. In addition, we compared newborn TREC content of all SCID patients reported in literature as a measure of diagnostic performance of different TREC cut-off values. We included articles discussing USA and non-USA data. By creating an overview of all available data, we aimed to strengthen the evidence for implementing NBS for SCID and to facilitate decision-making on available screening assays and possible algorithms.

## Methods

We conducted a systematic review on the diagnostic performance of TREC based NBS for SCID and reported our findings following the Preferred Reporting Items for Systematic Reviews and Meta-Analyses (PRISMA) guidelines [23].

### SCID Definitions

We divided reported cases into ‘typical SCID’ (defined as CD3 T cells < 300/μl or maternal engraftment) and ‘other forms of TCL’ (defined as CD3 T cells ≥ 300/μl) to match the diagnostic criteria for SCID that were recently established by the Primary Immune Deficiency Treatment Consortium using the data available [24]. In line with these criteria, we did not classify leaky SCID and Omenn syndrome as typical SCID. It was not possible to use the criteria to their full extent because none of the included publications reported on all parameters required. When no CD3 values were reported, we categorized the patients as reported by the authors.

### Literature Search

We searched PubMed, EMBASE (excl. MEDLINE) and the Cochrane library on June 20, 2014 for synonyms of “newborn screening” and “primary immunodeficiencies”. Our search strategy is shown in the Online Resource 1. We checked reference lists of all included studies for additional sources, including government reports. We contacted authors to obtain missing data and excluded studies with insufficient data to answer our research queries. During the analysis of the included studies, the largest original study on this topic was published; this study was included in this systematic review as well.

### Eligibility Criteria

We identified all studies evaluating TREC based NBS, detecting typical SCID and/or other causes of TCL. Both case series evaluating TRECs in stored DBS of more than two known TCL patients and cohort studies in which NBS TRECs were determined during the neonatal period, were eligible. We adopted no language restrictions.

### Study Selection

**Fig. 1:**
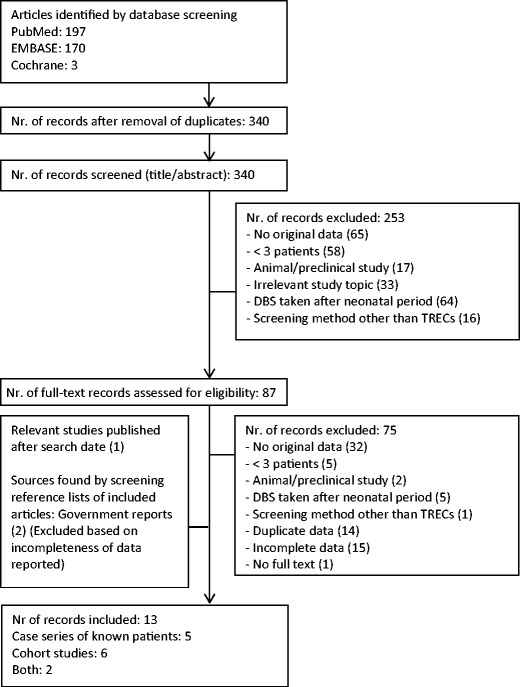
Flowchart of article selection for systematic review of TREC based NBS for SCID. Exclusion criteria were applied hierarchically. Studies were included as case series if stored Guthrie cards of known patients were tested. Studies were included as cohort studies if newborns were tested prospectively and sufficient details on algorithm outcome were reported

Our study selection process is shown in Fig. 1. We screened the titles and abstracts of all identified records and excluded articles in a standardized manner. The previously determined exclusion criteria were applied hierarchically (Fig. 1). Eligible articles, including those with an eligible title lacking an abstract, were assessed based on full text. If multiple records described the same screening population, we included the most recent publication. We included all case series stating which diagnoses would (not) have been detected by screening. Cohort studies without details on the TREC cut-off value and the number of re-tests, repeat DBSs and referrals were excluded.

### Data Extraction

The following items were extracted from all included studies: geographical location of the study, any parameters added to the screening panel in addition to TRECs, control gene, TREC cut-off value, number of newborns included in the study, number of re-tests on initial DBS specimens (defined as testing a second punch from the initial Guthrie card), number of repeat DBSs requested, number of referrals, number of typical SCID patients, number of other TCL patients, genetic diagnosis of confirmed typical SCID patients and diagnoses of other TCL patients. We assumed that presumptive positive newborns in pilot cohort studies would have been referred. Since not all studies reported PPVs and incidences, we calculated the PPV and incidence for SCID and TCL (typical SCID + other TCL) for all cohort studies based on the reported number of newborns, referrals and patients.

In addition, we extracted mean normal TREC content and TREC content of all newborns with a diagnosis that had been reported to feature TCL in any of the included studies when reported. Only TREC values derived from DBS taken at birth were included. To allow for comparison between studies we approximated that (TRECs/reaction)/3 equals TRECs/μl, since a 3.2 mm DBS punch is estimated to contain 3 μl of blood [13, 25, 26]. Notably, UK data were obtained using 1.5 mm punches but did not require conversion because data were reported in TRECs/μl. We deduced TREC content from graphs when exact content was not reported. If TREC content of patients was reported for initial and re-testing separately, we used mean TREC content.

### Assessment of Completeness of Reporting

We assessed the quality and completeness of reporting in the included studies. Since no standard for reporting screening studies without a reference standard exists, we developed assessment criteria based on the Standards for Reporting of Diagnostic Accuracy (STARD) guidelines [27]. Items reviewed are specified in Online Resource 2. In short, we assessed accuracy of reporting on the TREC cut-off value, number of retests, repeat DBSs and referrals, normal TREC content, cases identified, CD3 counts in cases identified, handling of loss to follow-up and the possibility of missed cases.

### Synthesis of Results

We created dot plots of TREC content and corresponding diagnoses. Additionally, we aimed to improve comparability between the different TREC assays and cut-off values by plotting the TREC content divided by the cut-off value against the diagnoses. Because of the heterogeneity of screening strategies, we did not perform diagnostic meta-analysis.

## Results

### Study Selection

Our search resulted in 340 unique records. After screening these, we included five case series [28–32], five cohort studies [12, 25, 26, 33, 34] and two studies describing both a case series and a cohort [13, 16] (Fig. 1). By checking the reference lists of included articles we identified two eligible government reports [11, 35]. However, these were excluded after full text screening because of incompleteness of data on the number of retests, repeat DBS and referrals and because complete data were presented in more recent publications (e.g. [33] for New York State). We included one extra study describing 11 newborn cohorts in the USA that was published after the search date of this review, but was considered important [36]. Four of these cohorts were partially published earlier [25, 26, 33, 34]. Since more data for data extraction were available in the earlier published studies, but more newborns were reported in the most recent study, we extracted data from earlier studies and the most recent study.

### Completeness of Reporting

We assessed the completeness of reporting in the included studies based on predefined review criteria (see Online Resource 2). According to these criteria, reporting was of high completeness in eleven studies (Online Resource 3). Data collection from the remaining studies [26, 36] was suboptimal, because of lacking data on retest, repeat DBS and referral rate, TREC values of individual patients and discussion of loss to follow-up.

### Results of Individual Studies

#### High Sensitivity for Typical SCID of TREC Based NBS in Case Series

TREC assays were validated by measuring TREC content in stored Guthrie cards of patients known to have SCID or another diagnosis that was hypothesized to be detected by TREC based NBS. In total 58 of 159 (36 %) patients included in these case series had typical SCID. Sensitivity of TREC based NBS in these cases was 100 % for all types of typical SCID tested (Table 1). Several other types of TCL such as delayed onset ADA-SCID could not be detected by TREC NBS. Interestingly, performing the kappa-deleting recombination excision circles (KREC) assay, which detects B cell lymphopenia [13, 37, 38], next to the TREC assay (described by Borte et al.) enabled detection of delayed-onset ADA-SCID in neonatal DBSs [39], as did tandem mass spectrometry [28]. Variations in control genes and cut-off values used did not influence the correct detection of SCID patients. Other diagnoses with an abnormal TREC screening reported in case series included DiGeorge/22q11 deletion syndrome and ataxia-telangiectasia (A-T) (Table 1).

**Table 1:** Study characteristics of case series identified by systematic review and diagnoses (not) detected by TREC assay in stored Guthrie cards of known patients

Author (year)	Ref	Location	Parameter	Cut-off	Nr screened	Diagnoses detected (N)	Diagnoses not detected (N)
Morinishi et al. (2009)	[31]	Japan	TREC	NR	15	ADA-SCID (2), IL2RG-SCID (10), JAK3-SCID (2), LIG4-SCID (1)	
Borte et al (2012)	[13]	Sweden	TREC + KREC	15 TRECs/μl 10 KRECs/μl	49	AK2-SCID (1), A-T (4), IL2RG-SCID (4), IL7RA-SCID (3), JAK3-SCID (2), NBS (2)^§^, RAG1-SCID (5), undefined SCID (3), XLA (4)^§^	
La Marca et al. (2013)	[28]	Italy	TREC	25 TRECs/ μl	8	Early onset ADA-SCID (3)	Delayed onset ADA-SCID (3)^a^, healthy ADA carriers (2)
Mallott et al. (2013)	[30]	USA California	TREC	25 TRECs/μl	13	A-T (7)	A-T (6)
Somech et al. (2013)	[29]	Israel	TREC + KREC	30 TRECs/3 mm punched disk 8 KRECs/3 mm punched disk	8	Complete DiGeorge (1), DCLRE1C-SCID (1), Omenn-SCID (2), RAG2-SCID (2), undefined SCID (1), XLA(1) ^b^	
Adams et al. (2014)	[16]	UK	TREC	40 TRECs/μl	18	ADA-SCID (4), IL2RG-SCID (2), Omenn-SCID (2), PNP-SCID (1), RAG-SCID (2), undefined SCID (7)	
Lingman et al. (2014)	[32]	Sweden	TREC + KREC	8 TRECs/μl 6 KRECs/μl	48 ^c^	22q11 deletion syndrome (8), complete DiGeorge (1),	22q11 deletion syndrome (39)

*NR*, not reported

^a^Patients would have been detected by tandem mass spectrometry

^b^TRECs normal, KRECs abnormal

^c^KRECs were normal in all patients

#### Variation in Technical Characteristics of Screening Assay and Algorithm used in Cohort Studies

Table 2 demonstrates the differences between the various TREC assays and algorithms used at different screening sites, as far as reported in included articles. Notably, TREC cut-off values varied from 7 to 252 TRECs/μl. Furthermore, Guthrie card punches used in the UK were 1.5 mm rather than the standard 3.2 mm as suggested by the EnLite neonatal TREC kit (Perkin Elmer) [16]. DNA elution/extraction methods were similar at all screening sites, with the exception of New York State, where a wash/red cell lysis buffer was used. Most USA states used singleplex assays, while multiplex assays were used outside the USA. The incorporation of a repeat DBS for prematurely born neonates or NICU patients instead of referral in case of an abnormal screen, and the addition of a borderline category at several screening sites, were the most important differences between screening algorithms.

**Table 2:** Characteristics of TREC assay and screening algorithm of cohort studies included in systematic review

		Borte et al. [13]	Adams et al. [16]	Audrain et al. [12]	Chien et al. [26]	Verbsky et al. [25] Wisconsin	Kwan et al. [34] California	Vogel et al. [33] New York State
TREC assay characteristics								
Punch size		3.2 mm ~3 μl blood	1.5mm^a^	3.2 mm	3.2 mm	3.2 mm	3.2 mm	3 mm
DNA elution/extraction method		Generation DNA elution solution QIAGEN	DNA elution	Washing & elution with S2	Generation DNA elution solution QIAGEN	Generation DNA purification solution and Generation DNA elution solution QIAGEN	DNA elution Generation DNA purification and elution solution QIAGEN	Wash/red cell lysis buffer
Platform quantitative PCR^b^		RT-qPCR	PCR – probe hybridization – Victor EnLite fluorometer	RT-qPCR	RT-qPCR	RT-qPCR	RT-qPCR (Light cycler)	RT-qPCR
PCR set-up		Triplex RQ-PCR: TREC/KREC/ACTB	TREC single plex	TREC/RNAseP multiplex	TREC singleplex	TREC single plex	TREC single plex	TREC single plex
Control gene		ACTB (β-actin)	ACTB (β-actin)	RNAseP	RNAseP	ACTB (β-actin)	ACTB (β-actin)	RNaseP
TREC cut-off value		15 TRECs/μl	15 TRECs/μl	100 (183) TRECs/μl	40 TRECs/μl	25 / 40 TRECs/μl	40TRECs/μl (25 TRECs/μl) in repeat test	125 (200) TRECs/μl
Positive control		Plasmid	Calibrator samples (consisted of three DBS calibrators, A-C, with known quantities of TREC and ß-actin DNA)	Plasmid	NR	Plasmid	Plasmid	Plasmid
Algorithm								
Initial TREC measurement without control gene		−	+	−	−	+	+	−
Re-testing new punch initial Guthrie card		+	+	+	−	+	+	+
Borderline category		−	−	+	−	−	−	+
Repeat DBS in case of insufficient control gene amplification		+	+	+	NR	+	+	NR
Repeat DBS in borderline category		NA	NA	NR	NA	NA	NA	+
Repeat DBS at 37weeks gestational age for premature with abnormal screen		−	−	−	−	+	−	+
Repeat DBS for NICU patients with abnormal screen		−	−	−	−	−	+	−
Immediate referral when low TREC and normal control upon singulate initial testing		−	NA	NR	+	NA	NA	+
	Kwan et al. [36] Colorado	Kwan et al. [36] Connecticut	Kwan et al. [36] Delaware	Kwan et al. [36] Massachusetts^f^	Kwan et al. [36] Michigan	Kwan et al. [36] Mississippi	Kwan et al. [36] Navajo Nation	Kwan et al. [36] Texas
TREC assay characteristics								
Punch size	NR	NR	NR	3.2 mm	NR	3.2 mm	3.2 mm	NR
DNA elution/extraction method	NR	NR	NR	Washing & elution with S2	NR	DNA elution Generation DNA purification and elution solution QIAGEN	DNA elution Generation DNA purification and elution solution QIAGEN	NR
Platform quantitative PCR^b^	NR	NR	NR	RT-qPCR	NR	RT-qPCR (Light cycler)	RT-qPCR (Light cycler)	NR
PCR set-up	NR	NR	NR	TREC/RNAseP multiplex	NR	TREC single plex	TREC single plex	NR
Control gene	ACTB (β-actin)	RNaseP	RNaseP	RPaseP	ACTB (β-actin)	ACTB (β-actin)	ACTB (β-actin)	RNaseP
TREC cut-off value	40 TRECs/μl	30/25 TRECs/μl^c^	16 (26) TRECs/μl	252 TRECs/μl	7 (11) TRECs/μl	40TRECs/μl (25 TRECs/μl) in repeat test	40TRECs/μl (25 TRECs/μl) in repeat test	150 (200)/110^c^
Positive control	NR	NR	NR	Plasmid	NR	Plasmid	Plasmid	NR
Algorithm								
Initial TREC measurement without control gene	+	−	−	−	−	+	+	+
Re-testing new punch initial Guthrie card	+	+	NR	+	−	+	+	+
Borderline category	−	+	+	−	+	−	−	+
Repeat DBS in case of insufficient control gene amplification	+	+	+	+	+	+	+	+
Repeat DBS in borderline category	NA	+	NR	NA	+	NA	NA	+
Repeat DBS at 37weeks gestational age for premature with abnormal screen	−	−	+^d^	−	−	−	−	−
Repeat DBS for NICU patients with abnormal screen	−	−	−	−	−	+	+	−
Immediate referral when low TREC and normal control upon singulate initial testing	NA	+ (if <10 TRECs/μl and control gene cycle threshold <28)	NR^e^	−	+	NA	NA	−

+ algorithm did involve item, − algorithm did not involve item, *NA* not applicable, *NR* not reported

^a^EnLite neotatal TREC kit (Perkin Elmer)

^b^RT-qPCR refers to TaqMan technology (Applied Biosystem)

^c^Lower cut-off for preterm infants

^d^DBS repeated at 38 weeks

^e^No cycle threshold – 3 TRECs: Alert, handling NR

^f^Assay and algorithm details reported in [40, 41]

#### TREC Cut-Off Value and Diagnostic Performance in Pilot Cohort Studies

Data were collected prospectively for approximately 13,000 newborns in pilot cohorts (Table 3). These studies demonstrated that lowering the TREC cut-off value from 40 to 20 TRECs/μl decreased the retest rate from 3.76 to 0.20 % and from 2.63 to 1.17 % in the UK and France, respectively, and referral rates decreased from 1.00 to 0.04 % (UK) and 0.18 to 0.04 % (France). In both countries the lower cut-off value (UK: 20 TRECs/μl, France: 100 TRECs/reaction) was recommended for future national screening programs. The first pilot with combined TREC and KREC screening was conducted in Sweden. Two of the presumptive positive newborns in this study (33 %) would be referred based on normal TRECs and abnormal KRECs, resulting in a total referral rate of 0.23 %. The repeat DBS rate ranged from 0.00 to 0.04 %. Because Guthrie cards were anonymized in the pilot studies, newborns with a positive screen were not actually referred and the outcome of confirmatory diagnostic testing was thus unknown.

**Table 3:** Characteristics and results of screening algorithms used for NBS for SCID in pilot and population-based screening cohorts identified by systematic review

Author (year)	Ref	Location	Para-meter	Cut-off	Nr screened	Nr retests	Nr re-peat DBS	Nr referrals	Nr SCID	Nr other TCL	PPV SCID (% (95 % CI))	PPV TCL (% (95 % CI))	Incidence SCID/100,000	Incidence TCL/100,000
Pilot studies														
Borte et al. (2012)	[13]	Sweden	TREC + KREC	15 TRECs/μl 10 KRECs/μl	2560	22 (0.86 %)	1 (0.04 %)	6 (0.23 %)	NA	NA	NA	NA	NA	NA
Adams et al. (2014)	[16]	UK	TREC	40 TRECs/μl	5081	191 (3.76 %)	1 (0.02 %)	51 (1.00 %)	NA	NA	NA	NA	NA	NA
				20 TRECs/ μl	5081	10 (0.20 %)	1 (0.02 %)	2 (0.04 %)	NA	NA	NA	NA	NA	NA
Audrain et al. (2014)	[12]	France	TREC	183 TRECs/reaction^a^	5028	132 (2.63 %)	2 (0.04 %)	9 (0.18 %)	NA	NA	NA	NA	NA	NA
				100 TRECs/reaction	5028	59 (1.17 %)	0 (0.00 %)	2 (0.04 %)	NA	NA	NA	NA	NA	NA
Population-based screening														
Chien et al. (2012)	[26]	Taiwan	TREC	40 TRECs/μl	106,391	0 (0.00 %)	432 (0.41 %)	24 (0.02 %)	2	16	8.3 (−2.7–19.4)	75.0 (57.7–92.3)	1.9	16.9
Verbsky et al. (2012)	[25]	USA Wisconsin	TREC	25/40 ^b^ TRECs/μl	207,696	449 (0.22 %)	292 (0.14 %)	72 (0.03 %)	2	31	2.8 (−1.0–6.6)	45.8 (34.3–57.3)	1.0	15.9
Kwan et al. (2014)	[36]	USA Wisconsin	TREC	25/40 ^b^ TRECs/μl	340,037	NR	NR	108 (0.03 %)	4	45	3.7 (1.0–7.3)	45.4 (36.0–54.8)	1.2	14.4
Kwan et al. (2013)	[34]	USA California	TREC	25 TRECs/μl	993,724	>897 (>0.09 %)	806 (0.08 %)	161 (0.02 %)	12	38	7.5 (3.4–11.5)	31.1 (23.9–38.2)	1.2	5.0
Kwan et al. (2014)	[36]	USA California	TREC	25 TRECs/μl	1,384,606	NR	NR	206 (0.01 %)	23	57	11.2 (6.9–15.5)	38.8 (32.2–45.5)	1.7	5.8
Vogel et al. (2014), Kwan et al. (2014)	[33, 36]	USA New York State	TREC	125 TRECs/μl^d^	485,912*	1745* (0.36 %)	1307 (0.27 %)	531 ^e^ (0.11 %)	9	88	1.7 (0.6–2.8)	18.3 (15.0–21.6)	1.9	20.0
Kwan et al. (2014)	[36]	USA Colorado	TREC	40 TRECs/μl	70,989	NR	NR	10 (0.01 %)	1	3	10.0 (−8.6–28.6)	40.0 (9.6–70.4)	1.4	5.6
Kwan et al. (2014)	[36]	USA Connecticut	TREC	30 TRECs/μl	57,136	NR	NR	22 (0.04 %)	3	6	13.6 (−0.7–28.0)	40.9 (20.4–61.5)	5.3	15.8
Kwan et al. (2014)	[36]	USA Delaware	TREC	16 TRECs/μl^f^	11,202	NR	NR	9 (0.08 %)	1	3	11.1 (−9.4–31.6)	44.4 (12.0–76.9)	8.9	35.7
Kwan et al. (2014)	[36]	USA Massachusetts	TREC	252 TRECs/μl	293,371	NR	NR	63 (0.02 %)	4	47	6.3 (0.3–12.4)	81.0 (71.3–90.6)	1.4	17.4
Kwan et al. (2014)	[36]	USA Michigan	TREC	7 TRECs/μl	162,528	NR	NR	114 (0.07 %)	2	76	1.8 (−0.7–4.2)	68.4 (59.9–77.0)	1.2	48.0
Kwan et al. (2014)	[36]	USA Mississippi	TREC	25 TRECs/μl	37,613	NR	NR	5 (0.01 %)	1	4	20.0 (−15.1–55.1)	100	2.7	13.3
Kwan et al. (2014)	[36]	USA Navajo Nation	TREC	25 TRECs/μl	3498	NR	NR	1 (0.03 %)	1	0	100	100	28.6	28.6
Kwan et al. (2014)	[36]	USA Texas	TREC	150 TRECs/μl	183,191	NR	NR	249 (1.35 %)	2	80	0.8 (−0.3–1.9)	32.9 (27.1–38.8)	1.1	44.8

* Screening results cover the period 2010-2012

*NA* not applicable, *NR* not reported

^a^Borderline category (39–183)

^b^The cut-off value was changed to 40 after 19 months of screening for the next 17 months of screening

^c^SCID cases reported by Kwan et al. 2014 [36] include typical SCID (*n* = 42), leaky SCID (CD3 300–1500, few naïve T cells) (*n* = 9) and Omenn syndrome (oligoclonal T cells) (*n* = 1)

^d^Borderline category (125–200)

^e^531 Patients were referred for further evaluation, in 478 this evaluation was completed

^f^Borderline category (17–26)

#### Diagnostic Performance of TREC Based NBS in Population-Based Cohorts Differs Across Studies

Twelve population-based cohorts on TREC based NBS for SCID were included, detecting a total of 53 typical SCID cases and 494 cases of other TCL in approximately 3.15 million screened newborns (Table 3). The incidence of typical SCID approximated 1.7:100,000. A high SCID incidence was reported in Connecticut, Delaware and the Navajo Nation. In the former two states, however, the incidence was not significantly different from other screening sites, because of the relatively low number of newborns screened [36]. The higher incidence in Navajo Nation is consistent with the known founder mutation in DCLRE1C [42].

Diagnostic performance differed across studies: retest rate ranged from 0.00 (Taiwan) to 0.36 % (New York State), repeat DBS rate from 0.08 (California) to 0.41 % (Taiwan), and referral rate from 0.01 % (California, Colorado, Mississippi) to 1.35 % (Texas). Notably, the algorithm used in Texas and Taiwan did not include retesting of the initial DBS. As compared to the pilot studies, population based studies had higher repeat DBS rates, whereas the number of referrals was lower.

Within the five largest cohorts, the PPV of the algorithms for typical SCID ranged from 0.8 % (95 % CI −0.3–1.9 %; Texas) to 11.2 % (6.9–15.5 %; California), and from 18.3 % (15.0–21.6 %; New York State) to 81.0 % (71.3–90.6 %; Massachusetts) for TCL (typical SCID + other TCL). Differences in outcome were accompanied by variation in the screening algorithms: TREC cut-off values adopted ranged from 25 TRECs/μl (California) to 252 TRECs/μl (Massachusetts). Massachusetts was the only one using a multiplex assay. In addition, handling of subgroups varied. Wisconsin and New York State added a repeat DBS prior to referral in neonates born < 37 of gestational age with an abnormal TREC screening. In contrast, California adopted a repeat DBS for neonatal intensive care unit (NICU) patients with an abnormal screen.

#### Diagnoses Detected in Population-Based Cohort Studies

The different types of typical SCID detected in the population-based cohort studies are displayed in Table 4. The most prevalent type was IL2RG-SCID (*n* = 10; 19 % of typical SCID cases), followed by IL7R-SCID (*n* = 6; 11 %), SCID of unknown etiology (*n* = 6; 11 %), ADA-SCID (*n* = 5; 9 %), RAG1-SCID (*n* = 5; 9 %), JAK3-SCID (*n* = 3; 6 %). Other forms of typical SCID were identified once. Unspecified TCL (*n* = 117), DiGeorge syndrome (*n* = 83) and cardiac anomalies (*n* = 30) were most common. Overall, the diagnoses detected in the cohort studies corresponded to those detected in case series of known patients (Table 1).

**Table 4:** Diagnoses (not) detected by NBS for SCID in population-based screening cohorts identified by systematic review

Author (year)	Ref	Location	Typical SCID detected by screening (N)	Other TCL detected by screening (N)	Diagnoses not detected by screening (N)
Chien et al. (2012)	[26]	Taiwan	IL2RG (1), RAG1 (1)	22q11.2 deletion syndrome (5), other medical conditions (9), variant SCID (2)	None reported
Kwan et al. (2014)	[36]	USA	IL2RG (9), IL7RA (6), ADA (5), RAG1 (4), JAK3 (3), DCLRE1C (1), RAG2 (1), CD3D (1), TC7A (1), Pallister-Killian syndrome with tetrasomy 12p (1), undefined (10^a^)	Leaky RAG1 (4^b^), leaky RMRP (2), leaky IL2RG (1), leaky DCLRE1 (1), leaky undefined (2), DiGeorge (78)^c^, trisomy 21 (21), AT (4), trisomy 18 (4), other syndromes with T cell impairment (29), cardiac anomalies (30), multiple congenital anomalies (23), other secondary T cell impairment (64), premature birth alone (29), variant SCID (12), unspecified TCL (117)	^d^Delayed onset ADA-SCID (1), MHC II deficiency (2), Wiskott-Aldrich syndrome (2)

^a^Genetic testing not completed in 4, for 6 patients no mutation was found with known SCID genes excluded

^b^1 patient with Omenn syndrome

^c^3 complete DiGeorge

^d^As reported in [34]

In line with the case series, a case of delayed onset ADA-SCID was missed by statewide TREC based NBS in California. None of the other cohort studies reported cases that were missed by screening. Notably, in Taiwan only part of the newborn population underwent NBS for SCID. Two additional SCID cases were identified after becoming symptomatic in the unscreened population. Both infants died. Retrospective analysis of TREC content in the Guthrie cards of these patients revealed undetectable TREC content, indicating that both would likely have been identified if screening would have been implemented nationwide [26].

### Synthesis of Results

Both pilot and population based cohort studies demonstrated the impact of the TREC cut-off value on the diagnostic performance of SCID screening algorithms. To establish whether absolute TREC content of identified patients also differed across studies, we extracted mean normal TREC content and the TREC content of individual cases reported in all case series and cohort studies included.

#### Normal TREC Content

We aimed to extract normal means and ranges of TREC content of different studies for optimal evaluation of TREC cut-off values. Mean normal TREC content, however, were only reported by three out of seven cohort studies (Online Resource 3). In the UK, mean normal TREC content was 119 TRECs/μl and median TREC content 101 TRECs/μl (range 0–1160). In Taiwan, mean TREC content was 283 TRECs/μl and median 202 TRECs/μl. In Wisconsin, mean normal TREC content was 225 TRECs/μl and median TREC content was 186 TRECs/μl (range <20–5184) [10], whereas mean normal TREC content was 1900 TRECs/μl [40] in Massachusetts, and 1832 TRECs/μl (95 % CI 1823–1841) in the New York State. The large variation in TREC cut-off values was thus accompanied by a wide spread in normal TREC content.

#### Individual Patient TREC Content

**Fig. 2:**
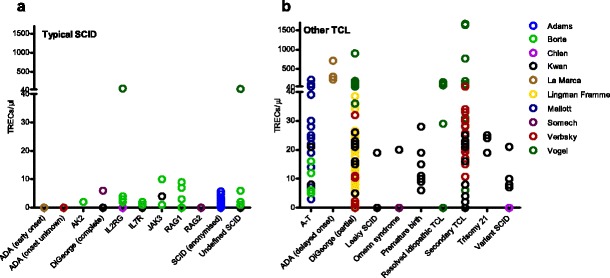
Neonatal TREC content of individual patients with typical SCID (**a**) and other TCL (**b**) diagnoses at different screening sites (*colors*). TREC content reported in TRECs/reaction was converted to TRECs/μl. Partial DiGeorge syndrome and 22q11 deletion syndrome are displayed together as DiGeorge (**b**). Thirty three of 74 typical SCID patients displayed had 0 TRECs, compared to 22 out of 182 other TCL patients. Anonimyzed SCID patients had ADA-SCID (*n* = 4), γ chain-SCID (*n* = 2), Omenn-SCID (*n* = 2), RAG-SCID (*n* = 2), PNP-SCID (*n* = 1), undefined SCID (*n* = 7)

We extracted the TREC content of all individual SCID/other TCL patients, as far as these were available in the included reports. Figure 2 shows the NBS TREC content plotted against the corresponding diagnoses. TREC content of typical SCID patients was lower than of patients with other causes of TCL. In fact, 37 of 80 SCID patients (46 %) displayed in Fig. 2 had undetectable TREC content, as compared to 18 of 172 non-SCID patients (11 %). Interestingly, higher TREC content was found in the patients reported by Vogel et al. [33]. This corresponds to the higher cut-off value used (Table 2) and the higher normal TREC content found in New York State. No difference existed between TREC values per diagnosis reported in case series and cohort studies.

**Fig. 3:**
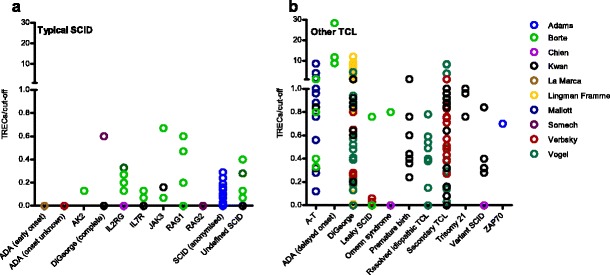
Neonatal TREC content of individual patients with typical SCID (**a**) and other TCL (**b**) diagnoses divided by screening site-specific TREC cut-off value. Colors correspond to different data sources

To compensate for the variation in cut-off values, we also plotted TREC content divided by cut-off value against the diagnoses (Fig. 3). By applying this calculation, all TREC values in SCID patients proved to be below the cut-off values, including the New York State cases. Cases in California were closest to the Californian stated cut-off value (TRECs/cut-off up to 0.8 in a patient with Omenn’s SCID).

**Fig. 4:**
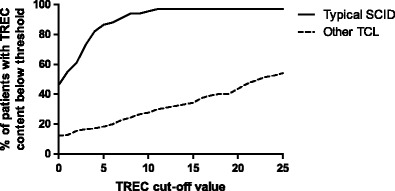
The percentage of typical SCID and other TCL cases of which the individual TREC score was below a TREC cut-off score from 0 to 25 TRECs/μl. Patients included in this figure are all patients for which an individual TREC score was reported in the included articles. Two typical SCID cases reported by Vogel et al. [33] had a TREC value above 25, as is consistent with the New York cut-off score of 200 TRECs/μl

Finally, we visualized the relation between of the cut-off score value and the percentage of typical SCID and other TCL cases identified by TREC screening in Fig. 4. Using a cut-off score of 25 TRECs/μl, approximately half of the other TCL cases reported in the included articles were detected.

## Discussion

In recent years, effective incorporation of TREC based NBS for SCID was reported in several studies. Techniques for measuring TRECs and specific screening and referral algorithms vary between states and countries that have reported successful implementation of SCID NBS. Case series of known patients confirmed the very high sensitivity of TREC based NBS for SCID, while prospective cohort studies confirmed the feasibility of screening to identify new SCID cases and significantly improve outcome of these patients. The reported incidence of SCID, although approximately twice the incidence reported before screening [43], was similar across screening sites, with the exception of Navajo Nation and states with a small cohort size; therefore, the major differences in PPV between the larger cohorts (SCID: 0.8–11.2 %, TCL: 18.3–81.0 %) must be explained by differences in screening algorithms.

To our knowledge, the present review is the most comprehensive review of TREC NBS so far, and it . is the first analysis of individual TREC content of patients identified by NBS at different screening sites. An overview of individual TREC content for SCID and other TCL patients provides new insight in the influence of the TREC cut-off value on screening efficacy. This systematic review provides an overview of test characteristics, screening algorithm details, and test performances, and thus facilitates comparing the different screening programs which can be of help for states and countries that are in the process of preparing for implementation of TREC based SCID screening. In the USA, NBS for SCID for >66 % of the annual newborn population was implemented in less than four years [7], demonstrating the speed with which SCID NBS can be implemented. The impact of SCID NBS will increase in the coming years, since multiple countries, including Canada [17], Brazil [14], the UK [16], France [12], Sweden [13], Germany [13] and also countries with reported high consanguinity rate and SCID incidence [44–46] are in various stages of implementing population-based SCID NBS.

A comprehensive overview of USA data exists was presented recently by Kwan et al. (2014). In the current review, we additionally provide the reported data from screening sites outside the US, demonstrating the value of recent developments such as the incorporation of screening for B cell lymphopenia by measurement of KREC content [13], as was proposed for incorporation in the USA as well [47]. Furthermore, this review reports the individual TREC values of typical SCID and other TCL patients detected by TREC-based NBS, which is of importance for decision making on identification of other causes of TCL that can be identified by TREC based NBS. Finally, we here report algorithm intermediates (re-tests and repeat DBS numbers) next to algorithm outcome (referral), as these numbers are of importance to calculate screening costs and burden put on individuals.

A decision tool was recently developed to calculate costs and benefits of SCID screening programs based on local birth rate, disease incidence and test costs. Assuming 100,000 births per year, an incidence of 1:33,000 and a cost price of 4.25 USD per test, SCID NBS was estimated to reduce SCID related healthcare costs from 6.0 to ~1.4 million USD annually in the USA [7]. The demonstrated cost-effectiveness [7, 8, 43], in combination with the clinical benefits, will likely stimulate other countries to also consider SCID NBS.

### Recommendations for the Development of SCID Screening Algorithms

Even though TREC assays were not uniform, individual TREC content of all SCID cases in both case series and cohort studies was < 25 TRECs/μl, except for two cases identified in New York State. Of the 74 typical SCID cases for whom TREC content was available, only the two New York cases would be missed using a cut-off of 20 TRECs/μl (total 2.7 %). 4.1 % would be missed with a cut-off of < 10 TRECs/μl. Lowering cut-off values will reduce the number of referrals and thereby the pressure exerted on the health care system, while not decreasing the sensitivity of NBS for SCID. Interestingly, the low TREC cut-off value (7 TRECs/μL) used in Michigan resulted in a relatively low PPV for typical SCID (1.8 %). The PPV for other TCL, however, was 68.4 %. This demonstrates that even with a low TREC cut-off value other non-SCID cases of significant TCL can be identified by NBS and also shows that not solely the TREC cut-off value, but also other factors in the PCR assay and algorithm determine PPV. Results of presently ongoing trials in Sweden and Germany using a cut-off value of <8 TRECs/μL and <4 KRECs/μL (reported in [48] and personal communication, Dr. S. Borte) will provide further insight on using low TREC cut-off values. New York State results deviated from other data, which may be explained by differences in the TREC assay used, e.g., procedure for DNA elution or qPCR conditions (Table 2). Lowering the TREC cut-off score will still result in identification of typical SCID cases and will reduce the number of other TCL cases identified. The cut-off level can thus be adapted to depending on the patient population that needs to be identified. Independent of the cut-off level that is used, a diagnostic infrastructure for further evaluation and clear guidelines for follow-up need to be established before implementation of TREC based SCID screening. This is especially true for cases of non-SCID TCL, because treatment and follow up of these cases is not always well defined

Adding KRECs next to TRECs to the screening panel allowed for the detection and thus early treatment of delayed onset ADA-SCID and primary immunodeficiencies featuring B cell lymphopenia (e.g., XLA, NBS [13] and possibly AT [49]). Future studies should reveal whether the benefits of early diagnosis of primary immunodeficiencies beyond SCID, as exemplified by the lower risk of chronic lung disease in XLA in cases diagnosed at young age [50], outweigh the increase in referral rate.

The highest PPV for typical SCID – but not TCL – was obtained by the Californian screening program that incorporated a repeat DBS for neonatal intensive care unit (NICU) patients with an abnormal screening report. Low TREC content in NICU patients has been confirmed in other studies [40, 41]. Several screening sites adopted a different cut-off for pre-term infants or added an extra TREC test at 37 weeks gestational age prior to referral, which lead to higher PPVs for TCL in Wisconsin and Delaware than in California. Studies have indicated a higher false positive rate in premature infants and lower mean TREC values [5, 10, 16, 25, 33]. Depending on whether new screening sites aim to detect typical SCID or also other causes of TCL, an extra TREC screen for NICU or premature infants should be considered as part of the algorithm.

Finally, to allow optimal comparison and to enable countries and states that newly implement TREC based NBS to design an evidence-based algorithm optimized for local use, we recommend the complete reporting of the results (including exact TREC values per identified case) of all screening sites.

### Limitations of Review and Published Studies

The inclusion of all raw screening data from all individual newborns reported in the included articles, and investigating the possibility of a universal cut-off value, would be ideal. These data, however, were not available; therefore, we used reported TREC content of cases and mean normal TREC content as the best available alternative. The level of detail provided on the diagnostic follow-up was not sufficient to allow us to fully apply the current definitions of SCID. We used CD3 levels and the vision of the authors of the articles instead. As a consequence, we could not separate leaky SCID from typical SCID cases in the USA cohorts.

Since population based NBS for SCID is mainly reported from the USA, the results presented in this review could reflect a selective sample. By including a cohort study from Taiwan, and several case series from other continents including Europe, Asia and South America, we increased the representativeness of the results. In fact, of 90 TREC content values of SCID patients, only 23 were from infants born in the USA. Results published so far however over-represent the Western countries. Future results on TREC based NBS from other regions, for instance those with higher consanguinity rates [45–47], may yield different data on incidence rates and on performance of the screening algorithms.

Reporting was not complete in most studies (Online Resource 3). For example, the study by Chien et al. lacked several of the items necessary for full description of screening performance, which indicates that the diagnostic performance found in this study should be considered with care. Importantly, no TREC values for detected cases were reported by Kwan et al. (2014). To allow comparison between future reports using different screening strategies and cut-off values we recommend that publications on screening cohorts will include at least a clear rationale for the cut-off value adopted, a flowchart with exact numbers of retests, repeat DBS and referrals, the mean normal TREC content in the population, details on the diagnosis of confirmed TCL patients, exact individual NBS TREC content for confirmed TCL patients, a description of patients who were lost to diagnostic follow-up, and a discussion of possibly missed cases.

## Conclusions

This systematic review of available literature provides an overview of screening performances of the different algorithms reported on TREC based SCID NBS. The compiled data demonstrate that variation in PCR assay and algorithm features are all determinants of the PPV of SCID NBS, the most important factor being the TREC cut-off value. The data in this review suggest that a using a TREC cut-off value of maximal 25 TRECs/μl and incorporating the collection of a repeat DBS from NICU patients with an abnormal screening results in the screening algorithm would be most effective in screening newborns for primary immunodeficiencies with TCL. This cut-off value would result in the detection of all typical SCID cases and the majority of other TCL cases; lowering the cut-off score would still lead to the identification of SCID cases, but reduce the amount of other TCL cases found. Incorporating an extra TREC test for premature infants prior to referral could increase the PPV for non-SCID TCL. New screening sites should therefore adjust their screening algorithm depending on the extent to which they want to identify non-SCID TCL. The addition of KREC based screening would allow identification of concurrent B cell lymphopenia, including patients with delayed-onset ADA-SCID, however a systematic evaluation of the effectiveness of additional KREC screening is not available yet.

## Electronic supplementary material

Below is the link to the electronic supplementary material.

ESM 1 (DOCX 106 kb)
