# Supplementary material for: TREC Based Newborn Screening for Severe Combined Immunodeficiency Disease: A Systematic Review
Source: J Clin Immunol. 2015 Apr 17;35(4):416–30. doi: 10.1007/s10875-015-0152-6 (PMC4438204; doi:10.1007/s10875-015-0152-6)
Supplement: Supplementary file 1 — (DOCX 106 kb) [file 10875_2015_152_MOESM1_ESM.docx]

**Electronic Supplementary Material for ‘TREC Based Newborn Screening for Severe Combined Immunodeficiency Disease: A Systematic Review’**

**Journal of Clinical Immunology**

Jet van der Spek, BSc^a^

Rolf H. H. Groenwold, MD, PhD^b^

Mirjam van der Burg, PhD^c^

Joris M. van Montfrans, MD, PhD^a^

^a^ Department of Pediatric Immunology and Infectious Diseases, University Medical Center Utrecht, Utrecht, The Netherlands

^b^ Julius Center for Health Sciences and Primary Care, University Medical Center Utrecht, Utrecht, The Netherlands

^c^ Department of Immunology, Erasmus MC, University Medical Center Rotterdam, Rotterdam, The Netherlands

Corresponding author:

Joris M. van Montfrans, MD, PhD

University Medical Center Utrecht

Lundlaan 6, PO Box 85090, 3508 AB Utrecht, The Netherlands

Tel: +31 (0)88 755 4003

Fax: +31 (0)88 755 5030

j.vanmontfrans@umcutrecht.n

**ONLINE RESOURCE 1**

**Search strategy (conducted on June 20, 2014)**

PubMed

#1 (17056 hits)

“Newborn screening”[tiab] OR “Neonatal screening”[tiab] OR (“Newborn”[tiab] AND “Screening”[tiab]) OR (“Neonatal”[tiab] AND “Screening”[tiab]) OR “Guthrie card”[tiab] OR “Guthrie test”[tiab] OR “Dried blood spot”[tiab] OR “Dried blood spots”[tiab] OR “T cell receptor excision circle”[tiab] OR “T cell receptor excision circles”[tiab] OR “T-cell receptor excision circle”[tiab] OR “T-cell receptor excision circles”[tiab] OR “TREC”[tiab] OR “TRECs”[tiab] OR “Kappa deleting recombination excision circle”[tiab] OR “Kappa deleting recombination excision circles”[tiab] OR “Kappa-deleting recombination excision circle”[tiab] OR “Kappa-deleting recombination excision circles”[tiab] OR “KREC”[tiab] OR “KRECs”[tiab] OR “Neonatal screening”[MeSH] OR “Dried Blood Spot Testing”[MeSH]

#2 (30605 hits)

“PID”[tiab] OR “primary immune deficiency”[tiab] OR “primary immune deficiencies”[tiab] OR “primary immunodeficiency”[tiab] OR “primary immunodeficiencies”[tiab] OR “SCID”[tiab] OR “Severe combined immunodeficiency”[tiab] OR “T cell lymphopenia”[tiab] OR “T cell lymphopenias”[tiab] OR “T-cell lymphopenia”[tiab] OR “T-cell lymphopenias”[tiab] OR “XLA”[tiab] OR “X linked agammaglobulinemia”[tiab] OR “X-linked agammaglobulinemia”[tiab] OR “22q11 deletion syndrome”[tiab] OR “DiGeorge syndrome”[tiab] OR “ataxia telangiectasia”[tiab] OR “ataxia-telangiectasia”[tiab] OR “Nijmegen Breakage Syndrome”[tiab] OR “Nijmegen-breakage-syndrome”[tiab] OR “B cell lymphopenia”[tiab] OR “B cell lymphopenias”[tiab] OR “B-cell lymphopenia”[tiab] OR “B cell lymphopenias”[tiab] OR “B cell maturation defect”[tiab] OR “B cell maturation defects”[tiab] OR “B-cell maturation defect”[tiab] OR “B-cell maturation defects”[tiab] OR “Severe Combined Immunodeficiency”[MeSH] OR “Bruton type agammaglobulinemia”[Supplementary Concept] OR “22q11 Deletion Syndrome”[MeSH] OR “DiGeorge Syndrome”[MeSH] OR “Ataxia Telangiectasia”[MeSH] OR “Nijmegen Breakage Syndrome”[MeSH]

#3 (197 hits)

#1 AND #2

EMBASE

#1 (16584 hits)

‘Newborn screening’:ti,ab OR ‘Neonatal screening’:ti,ab OR ‘Guthrie card’:ti,ab OR ‘Guthrie test’:ti,ab OR ‘Dried blood spot’:ti,ab OR ‘Dried blood spots’:ti,ab OR ‘T cell receptor excision circle assay’:ti,ab OR ‘T cell receptor excision circles’:ti,ab OR ‘T-cell receptor excision circle assay’:ti,ab OR ‘T-cell receptor excision circles’:ti,ab OR TREC:ti,ab OR TRECs:ti,ab OR ‘Kappa deleting recombination excision circle’:ti,ab OR ‘Kappa deleting recombination excision circles’:ti,ab OR ‘Kappa-deleting recombination excision circle’:ti,ab OR ‘Kappa-deleting recombination excision circles’:ti,ab OR KREC:ti,ab OR KRECs:ti,ab OR ‘Newborn screening’/exp OR ‘Dried Blood Spot Testing’/exp

#2 (42391 hits)

‘PID’:ti,ab OR ‘primary immune deficiency’:ti,ab OR ‘primary immune deficiencies’:ti,ab OR ‘primary immunodeficiency’:ti,ab OR ‘primary immunodeficiencies’:ti,ab OR ‘SCID’:ti,ab OR ‘Severe combined immunodeficiency’:ti,ab OR ‘T cell lymphopenia’:ti,ab OR ‘T cell lymphopenias’:ti,ab OR ‘T-cell lymphopenia’:ti,ab OR ‘T-cell lymphopenias’:ti,ab OR ‘XLA’:ti,ab OR ‘X linked agammaglobulinemia’:ti,ab OR ‘X-linked agammaglobulinemia’:ti,ab OR ‘22q11 deletion syndrome’:ti,ab OR ‘DiGeorge syndrome’:ti,ab OR ‘ataxia telangiectasia’:ti,ab OR ‘ataxia-telangiectasia’:ti,ab OR ‘Nijmegen Breakage Syndrome’:ti,ab OR ‘Nijmegen-breakage-syndrome’:ti,ab

OR ‘B cell lymphopenia’:ti,ab OR ‘B cell lymphopenias’:ti,ab OR ‘B-cell lymphopenia’:ti,ab OR ‘B cell lymphopenias’:ti,ab OR ‘B cell maturation defect’:ti,ab OR ‘B cell maturation defects’:ti,ab OR ‘B-cell maturation defect’:ti,ab OR ‘B-cell maturation defects’:ti,ab OR ‘Severe Combined Immunodeficiency’/exp OR ‘X linked agammaglobulinemia’/exp OR ‘chromosome deletion 22q11’/exp OR ‘DiGeorge syndrome’/exp OR ‘Ataxia Telangiectasia’/exp OR ‘Nijmegen Breakage Syndrome’/exp

#3 (170 hits)

#1 AND #2 AND [embase]/lim NOT [medline]/lim

Cochrane

#1 (596 hits)

"Newborn screening" or "Neonatal screening" or ("Newborn" and "Screening") or ("Neonatal" and "Screening") or "Guthrie card" or "Guthrie test" or "Dried blood spot" or "Dried blood spots" or "T cell receptor excision circle" or "T cell receptor excision circles" or "T-cell receptor excision circle" or "T-cell receptor excision circles" or "TREC" or "TRECs" or "Kappa deleting recombination excision circle" or "Kappa deleting recombination excision circles" or "Kappa-deleting recombination excision circle" or "Kappa-deleting recombination excision circles" or "KREC" or "KRECs":ti or "Newborn screening" or "Neonatal screening" or ("Newborn" and "Screening") or ("Neonatal" and "Screening") or "Guthrie card" or "Guthrie test" or "Dried blood spot" or "Dried blood spots" or "T cell receptor excision circle" or "T cell receptor excision circles" or "T-cell receptor excision circle" or "T-cell receptor excision circles" or "TREC" or "TRECs" or "Kappa deleting recombination excision circle" or "Kappa deleting recombination excision circles" or "Kappa-deleting recombination excision circle" or "Kappa-deleting recombination excision circles" or "KREC" or "KRECs":ab (Word variations have been searched)

#2 (470 hits)

"PID" or "primary immune deficiency" or "primary immune deficiencies" or "primary immunodeficiency" or "primary immunodeficiencies" or "SCID" or "Severe combined immunodeficiency" or "T cell lymphopenia" or "T cell lymphopenias" or "T-cell lymphopenia" or "T-cell lymphopenias" or "XLA" or "X linked agammaglobulinemia" or "X-linked agammaglobulinemia" or "22q11 deletion syndrome" or "DiGeorge syndrome" or "ataxia telangiectasia" or "ataxia-telangiectasia" or "Nijmegen Breakage Syndrome" or "Nijmegen-breakage-syndrome" or "B cell lymphopenia" or "B cell lymphopenias" or "B-cell lymphopenia" or "B cell lymphopenias" or "B cell maturation defect" or "B cell maturation defects" or "B-cell maturation defect" or "B-cell maturation defects" or "Bruton type agammaglobulinemia":ti or "PID" or "primary immune deficiency" or "primary immune deficiencies" or "primary immunodeficiency" or "primary immunodeficiencies" or "SCID" or "Severe combined immunodeficiency" or "T cell lymphopenia" or "T cell lymphopenias" or "T-cell lymphopenia" or "T-cell lymphopenias" or "XLA" or "X linked agammaglobulinemia" or "X-linked agammaglobulinemia" or "22q11 deletion syndrome" or "DiGeorge syndrome" or "ataxia telangiectasia" or "ataxia-telangiectasia" or "Nijmegen Breakage Syndrome" or "Nijmegen-breakage-syndrome" or "B cell lymphopenia" or "B cell lymphopenias" or "B-cell lymphopenia" or "B cell lymphopenias" or "B cell maturation defect" or "B cell maturation defects" or "B-cell maturation defect" or "B-cell maturation defects" or "Bruton type agammaglobulinemia":ab (Word variations have been searched)

#3 (3 hits)

#1 AND #2

**ONLINE RESOURCE 2**

**Completeness of reporting assessment protocol for included studies**

**Assessment items per domain:**

1. Methods
   1. Describes TREC assay (incl. control gene)
   2. Reports TREC unit clearly
   3. Reports TREC cut-off value
   4. Reports rationale for cut-off
2. Results
   1. Reports number of re-tests precisely
   2. Reports number of repeat DBSs precisely
   3. Reports number of referrals precisely
   4. Reports mean normal TREC content
   5. Reports exact TREC content for newborns with TCL
   6. Reports CD3 values for identified cases
   7. Reports genetic diagnoses of individual typical SCID patients with TREC content
   8. Reports diagnosis of individual other TCL patients with TREC content
   9. Reports how many newborns referred did were lost to diagnostic follow-up
   10. Describes why newborns were lost to follow-up
3. Discussion
   1. Discusses whether cases could have been missed

**Assessment protocol**

1. Case series were assessed on items 1a-d, 2d-g. Pilot cohort studies were assessed on items 1a-d, 2a-d. Other items were considered non applicable (NA). If only typical SCID patients were tested item 2g was NA. If only other TCL patients were tested item 2f was NA. Population-based cohort studies were assessed on all items.
2. All questions were answered with yes (**X**) or no (**O**).
3. If more than half of applicable items within methods, results or discussion was scored as ‘no’, the study was considered to have low completeness of reporting.

| Table 5. Assessment of completeness of reporting in studies included in systematic review based on adjusted STARD criteria | | | | | | | | | | | | | | | | | | | | | | | | | | | | | | | | | | | |  | |  |
| --- | --- | --- | --- | --- | --- | --- | --- | --- | --- | --- | --- | --- | --- | --- | --- | --- | --- | --- | --- | --- | --- | --- | --- | --- | --- | --- | --- | --- | --- | --- | --- | --- | --- | --- | --- | --- | --- | --- |
|  | | **Methods** | | | | |  | | | | **Results** | | | | | | | | | | | | | | | | | | | | | | | | **Discussion** | |  | |
|  | | **TREC assay** | **TREC unit** | **TREC cut-off** | | **Rationale cut-off** | **Nr. retests** | | **Nr. repeat DBS** | | | **Nr. referrals** | | **Mean normal TREC content** | | **Exact TREC content** | | | | **CD3 values** | | | **Genetic diagnosis SCID** | | | **Diagnoses other TCL** | | | **Nr. loss to follow-up** | | | **Reason loss to follow-up** | | | **Missed cases** | | **Overall completness** | |
| Case series | | |  | |  |  | |  | |  | | |  | |  | | |  | | |  | | |  | | |  | | |  | | |  | | | |  | |
| Morinishi | | **X** | **X** | **O** | | **O** | NA | | NA | | | NA | | **X** | | | **X** | | **X** | | | **X** | | | NA | | | NA | | | NA | | | NA | | | high | |
| Borte | | **X** | **X** | **X** | | **X** | NA | | NA | | | NA | | **O** | | | **O** | | **O** | | | **X** | | | **X** | | | NA | | | NA | | | NA | | | high | |
| La Marca | | **X** | **X** | **X** | | **O** | NA | | NA | | | NA | | **O** | | | **X** | | **O** | | | **X** | | | NA | | | NA | | | NA | | | NA | | | high | |
| Mallott | | **X** | **X** | **X** | | **O** | NA | | NA | | | NA | | **O** | | | **X** | | **X** | | | NA | | | **X** | | | NA | | | NA | | | NA | | | high | |
| Somech | | **X** | **X** | **X** | | **X** | NA | | NA | | | NA | | **X** | | | **X** | | **X** | | | **X** | | | NA | | | NA | | | NA | | | NA | | | high | |
| Adams | | **X** | **X** | **X** | | **X** | NA | | NA | | | NA | | **X** | | | **X** | | **O** | | | **O** | | | NA | | | NA | | | NA | | | NA | | | high | |
| Audrain | | **X** | **X** | **X** | | **X** | NA | | NA | | | NA | | **O** | | | **X** | | **O** | | | **X** | | | NA | | | NA | | | NA | | | NA | | | high | |
| Lingman | | **X** | **X** | **X** | | **X** | NA | | NA | | | NA | | **O** | | | **X** | | **X** | | | NA | | | **X** | | | NA | | | NA | | | NA | | | high | |
| Cohort studies | | |  | |  |  | |  | |  | | |  | |  | | |  | | |  | | |  | | |  | | |  | | |  | | | |  | |
| Borte | | **X** | **X** | **X** | | **X** | **X** | | **X** | | | **X** | | **O** | | | NA | | NA | | | NA | | | NA | | | NA | | | NA | | | NA | | | high | |
| Adams | | **X** | **X** | **X** | | **X** | **X** | | **X** | | | **X** | | **X** | | | NA | | NA | | | NA | | | NA | | | NA | | | NA | | | NA | | | high | |
| Audrain | | **X** | **X** | **X** | | **X** | **X** | | **X** | | | **X** | | **O** | | | NA | | NA | | | NA | | | NA | | | NA | | | NA | | | NA | | | high | |
| Chien | | **X** | **X** | **X** | | **X** | **O** | | **O** | | | **O** | | **X** | | | **X** | | **X** | | | **X** | | | **X** | | | **O** | | | **O** | | | **X** | | | low | |
| Verbsky | | **X** | **X** | **X** | | **X** | **X** | | **X** | | | **X** | | **O** | | | **X** | | **X** | | | **X** | | | **X** | | | **O** | | | **O** | | | **X** | | | high | |
| Kwan (2013) | | **X** | **X** | **X** | | **O** | **O** | | **X** | | | **X** | | **O** | | | **X** | | **X** | | | **X** | | | **X** | | | **X** | | | **X** | | | **X** | | | high | |
| Kwan (2014) | | **X** | **X** | **X** | | **O** | **O** | | **O** | | | **X** | | **O** | | | **O** | | **O** | | | **X** | | | **X** | | | **O** | | | **O** | | | **X** | | | low | |
| Vogel | | **X** | **O** | **X** | | **X** | **X** | | **X** | | | **X** | | **X** | | | **X** | | **X** | | | **X** | | | **X** | | | **X** | | | **X** | | | **X** | | | high | |
| X, item reported; O, item not reported (for specification of individual items see Online Resource 2); NA, not applicable | | | | | | | | | | | | | | | | | | | | | | | | | | | | | | | | | | | |  | |  |
|  |  | | | | | | | | | | | | | | | | | | | | | | | | | | | | | | | | | | |  | |  |

**ONLINE RESOURCE 3**
